# Minnesota State Veterinary Medical Association

**Published:** 1900-02

**Authors:** K. J. McKenzie

**Affiliations:** Secretary-Treasurer


Secretary.

### MINNESOTA STATE VETERINARY MEDICAL ASSOCIATION.

The fifth semi-annual meeting of the Minnesota State Veterinary Medical Association was held at the Merchants' Hotel, in St. Paul, January 11 and 12, 1900. President Dr. M. H. Reynolds, of St. Anthony Park, called the meeting to order.

The following members responded to roll-call: Drs. C. C. Lyford, S. D. Brimhall, J. G. Annand, J. S. Butler, A. A. Keyes, and M. J. Sexton, of Minneapolis; B. A. Pomeroy, R. Price, and G. A. Dallamore, of St. Paul; M. H. Reynolds, of St. Anthony Park; J. P. Anderson, of Rochester; L. Hay, of Faribault; K. J. McKenzie, of Northfield; B. Lambrechts, of Montevideo; S. H. Ward, of St. Cloud; W. Amos, of Owatonna; J. N. Gould, of Worthington; George McGillivary, of Pipestone; J. N. Gould, of Fairmont; J. J. McLaughlin, of Blue Earth City; H. C. Lyons, of Hutchinson; H. C. Peters, of Litchfield; F. H. Farmer, of Wahpeton, N. D.

President Reynolds gave an address, speaking lengthily on how to make a State Veterinary Medical Association a success, bringing out some very useful points with regard to place and way of holding Association meetings, emphasizing the fact that Association work should be based upon the work of its individual members.

Dr Ward gave a report on veterinary college education, bringing out some very important facts in regard to graduates of two-year colleges.

Dr. Reynolds, as Chairman of the Committee on Recent Veterinary Literature, read a very interesting report on the great advances made in veterinary surgery.

Dr. Brimhall reported some results obtained from the use of potassium iodide in the treatment of parturient paresis and in azoturia. Dr. Brimhall also spoke quite lengthily on dealing with infectious diseases in animals.

Dr. C. C. Lyford then made his report as Chairman of the Committee on Legislation and Empirics, stating that the case against Dr. McCullock, of Minneapolis, for illegal practice had been set for trial on January 19th, he having pleaded not guilty.

Farmer Miles, of Charleston, Ill., who was present at the meeting, was called upon and made some very interesting remarks on ridgling castration.

The meeting then adjourned to the State Farm for supper, after which clinics, as follows:

Removal of Scirrhous Cord, Farmer Miles; Operation for Fistulous Withers, Dr. J. N. Gould; Removal of Lateral Cartilage for Quittor, Dr. C. C. Lyford.

The meeting then adjourned till 9 A.M., Friday, 12th, when the following applicants were taken in as new members of the Association: Dr. C. T. Eckles, St. Charles; W. A. Sprouler, Waseca; M. S. Whitcomb, Austin; Dr. E. T. Frank, Warren; T. Falconer, Glennwood.

On motion, Drs. Reynolds and Brimhall were appointed to draw up an agreement with the Board of Trustees of the State Agricultural College to make arrangements for a Minnesota State Veterinary Medical Museum, to be contributed to by the different members of the Association.

The Secretary then read a circular letter from Dr. Salmon, asking for the support of the individual members in fighting for the bill for the improvement of the United States Army Veterinary Corps.

On motion, the President appointed a committee of three to draw up resolutions representing the sentiments of the Association with regard to the Army bill.

Committee: Dr. S. H. Ward, Dr. E. T. Frank, Dr. J. G. Annand.

A voluntary contribution was then taken up and the Secretary instructed to send the same to Dr. Salmon for the U. S. V. M. A. Committee on Army Legislation.

The election of officers then took place with the following result: President, Dr. S. H. Ward; First Vice-President, Dr. W. Amos; Second Vice-President, Dr. C. C. Lyford; Secretary and Treasurer, Dr. K. J. McKenzie. Trustees: Drs. B. Lambrecht, H. C. Lyons, and J. S. Butler.

The meeting then adjourned for dinner.

At 2 P.M. the meeting was called to order by President S. H. Ward.

Dr. C. C. Lyford reported a case of unusual and interesting lesions of glanders, and exhibited a portion of a trachea showing marked glanderous lesions.

A very interesting discussion followed on the use of mallein in the diagnosis of glanders.

Dr. Head then read a very interesting paper on the "Leucocyte Count in Septic Infection," followed by an interesting discussion.

On motion, a vote of thanks was tendered Dr. Head for the manner in which he handled his subject.

The following committees were then appointed: Colleges—Dr. S. H. Ward. Infectious Diseases—Dr. S. D. Brimhall. Finances—Dr. M. J. Sexton. Legislation—Drs. J. S. Butler, R. Price, and C. C. Lyford.

The Committee on Recent Veterinary Literature was subdivided as follows: Surgery—Dr. M. H. Reynolds. Bacteriology—Dr. S. D. Brimhall. Medicine—Dr. J. N. Gould.

The meeting then adjourned.

K. J. McKenzie,
Secretary-Treasurer.

